# Impact of home care versus alternative locations of care on elder health outcomes: an overview of systematic reviews

**DOI:** 10.1186/s12877-016-0395-y

**Published:** 2017-01-14

**Authors:** Laura Boland, France Légaré, Maria Margarita Becerra Perez, Matthew Menear, Mirjam Marjolein Garvelink, Daniel I. McIsaac, Geneviève Painchaud Guérard, Julie Emond, Nathalie Brière, Dawn Stacey

**Affiliations:** 1 Population Health, Faculty of Health Sciences, University of Ottawa, 25 University Private, Ottawa, ON K1N 7 K4 Canada; 2 CHU de Québec Research Centre-Université Laval site Hôpital St-Francois d’Assise, 10 Rue Espinay, Quebec, G1L 3 L5 Canada; 3 Department of Anesthesiology, Faculty of Medicine University of Ottawa, The Ottawa Hospital, 1053 Carling Ave, Rm B311, Ottawa, ON K1Y 4E9 Canada; 4 Centre de santé et de services sociaux de la Vieille-Capitale, 880, rue Père-Marquette, Québec, G1M 2R9 Canada; 5 Centre intégré universitaire en santé et services sociaux de la Capitale-Nationale, 880, rue Père-Marquette, Québec, G1M 2R9 Canada; 6 ﻿Ottawa Hospital Research Institute, 501 Smyth Road, Ottawa, ON K1H 8L6 Canada; 7 University of Ottawa, 451 Smyth Road, Ottawa, ON K1H 8 M5 Canada

**Keywords:** Location of elderly care, Health, Overview of reviews, Shared decision making, Decision Support

## Abstract

**Background:**

Many elders struggle with the decision to remain at home or to move to an alternative location of care. A person’s location of care can influence health and wellbeing. Healthcare organizations and policy makers are increasingly challenged to better support elders’ dwelling and health care needs. A summary of the evidence that examines home care compared to other care locations can inform decision making. We surveyed and summarized the evidence evaluating the impact of home care versus alternative locations of care on elder health outcomes.

**Methods:**

We conducted an overview of systematic reviews. Data sources included MEDLINE, the Cochrane Library, EMBASE, and CINAHL. Eligible reviews included adults 65+ years, elder home care, alternative care locations, and elder health outcomes. Two independent reviewers screened citations. We extracted data and appraised review quality using the Assessing the Methodological Quality of Systematic Reviews (AMSTAR) checklist. Results were synthesized narratively.

**Results:**

The search yielded 2575 citations, of which 19 systematic reviews were eligible. Three hundred and forty studies with 271,660 participants were synthesized across the systematic reviews. The categories of comparisons included: home with support versus independent living at home (*n* = 11 reviews), home care versus institutional care (*n* = 3 reviews), and rehabilitation at home versus conventional rehabilitation services (*n* = 7 reviews). Two reviews had data relevant to two categories. Most reviews favoured home with support to independent living at home. Findings comparing home care to institutional care were mixed. Most reviews found no differences in health outcomes between rehabilitation at home versus conventional rehabilitation services. Systematic review quality was moderate, with a median AMSTAR score of 6 (range 4 - 10 out of 11).

**Conclusions:**

The evidence on the impact of home care compared to alternative care locations on elder health outcomes is heterogeneous. Our findings support positive health impacts of home support interventions for community dwelling elders compared to independent living at home. There is insufficient evidence to determine the impact of alternative care locations on elders’ health. Additional research targeting housing and care options for the elderly is needed.

**Electronic supplementary material:**

The online version of this article (doi:10.1186/s12877-016-0395-y) contains supplementary material, which is available to authorized users.

## Background

Location of care (LOC) for the elderly has become an increasingly important societal issue [1]. With a demographic shift towards an aging population with complex healthcare needs, healthcare systems face challenges in providing long-term care for elders [2, 3]. One such challenge is matching the elders’ health and wellbeing needs to the environment. The ecological theory of aging suggests that the environment influences elders’ functional status. Promoting optimal outcomes requires a goodness of fit between the elders and the environment [4]. For example, the person-environment fit can positively or negatively influence health outcomes if personal competencies are well-suited, or alternatively, poorly matched to environmental demands. The World Health Organization’s report on Aging and Health highlighted the importance of environmental influence on functioning and research has shown that elders’ opportunity to build and maintain functional ability is enhanced when they live in an environment that addresses their needs [5]. As such, decision makers (e.g., policy makers, patients, family members, and healthcare providers) are challenged to better consider the impact of the environment, or LOC, on elders’ health and wellbeing outcomes.

To make a well-informed decision, stakeholders need to know their options (i.e., available LOCs), the benefits and harms of each option, and how these benefits and harms relate to their personal situation [6]. Common long-term LOC options include home care and institutional care [7]. Home care typically includes independent living at home or living at home with supports and/or modifications to enhance health and independence. Institutional LOCs typically refer to nursing home care or skilled nursing care facilities. Elders and caregivers have identified deciding about moving from home to an alternative LOC as one of the most difficult decisions they face [8, 9]. This decision is complicated by the evolving contextual factors relating to the care situation such as elder health status, characteristics of the caregivers, and physical environment [8]. Access to evidence describing the impact of home care versus an alternative LOC on elder health and wellbeing could help inform decision making.

Several systematic reviews have examined the impact of LOC on elder health outcomes. However, these reviews tend to narrowly focus on the health impact of a specific intervention (e.g., fall reduction interventions, palliative care, case management) delivered at different care locations. Although individual reviews provide important contributions, a single document that summarizes a broader range of findings will improve access to the literature and help inform stakeholders’ decisions about elder LOC. Additionally, it will highlight important knowledge gaps and help prioritise future research questions. Therefore, we summarized available research findings that examined the impact of home care versus alternative LOCs on elder health and well-being.

## Methods

### Design

We conducted an overview of systematic reviews. An overview design attempts to survey, summarize, and describe the literature allowing findings to be efficiently compared and contrasted [10]. The paper is reported according to the Preferred Reporting Items for Systematic Reviews and Meta-Analyses (PRISMA) guidelines [11]. However, we did not register our protocol in PROSPERO [12]. We initially piloted our protocol using a single database [13]. Changes from the original protocol to the current study included limiting the population from elders and caregivers, to elders only. Our protocol can be available to readers upon request to the corresponding author.

### Review eligibility criteria

The PICOS framework guided systematic review eligibility criteria (Table 1). We included systematic reviews that examined: adults aged 65 or greater, home care, alternative LOCs, and health outcomes (e.g., physical and mental health, morbidity, mortality, functional status and dependence, activities of daily living, quality of life, falls, etc.). We defined systematic reviews as a review of the evidence that included a clear research question, used systematic methods to identify, select and appraise the primary research, and extracted and analysed/synthesized data from included studies [14]. We did not restrict systematic reviews types (e.g. meta-analysis, narrative), dates, or language of publication. Non-systematic reviews, individual studies, and abstracts were excluded. Participant’s age had to be reported in the results section of the systematic review to be included. When systematic reviews reported a broad age range, we included the review if the mean participant age was 65 years or greater and/or if a sub-group analysis was conducted on participants with a mean age of 65 years or greater. Although our focus was specific to LOCs (i.e., home or alternative setting), we included reviews that examined interventions, care options, and service delivery models within the context of eligible LOCs. Ineligible LOCs were acute hospital stays and temporary or transitional placements (e.g., respite care or community/institutional placements for less than 3 months) [15]. If two reviews had 100% overlap of included studies (e.g., systematic review updates, systematic reviews with the same aims and outcomes), we included the review with the most complete and up-to-date dataset. All eligible LOC comparisons were considered.

**Table 1:** Inclusion and Exclusion Criteria

Criteria	Included	Excluded
Population	Adults 65 years old or greater	
Interventions	Home care	Acute hospital stay Respite or short term stay (<3 months)
Comparison	Alternative long-term LOCs	Acute hospital stay Respite or short term stay (<3 months)
Outcomes	Elders' health and wellbeing outcomes, including but not limited to: physical and physiological health, morbidity, mortality, functional status and dependence, activities, quality of life, and falls.	
Design	Systematic reviews, with or without meta-analyses, of studies that reviewed randomised controlled trials, controlled clinical trials, controlled before and after studies, descriptive studies, cohort studies, retrospective studies, and cross-sectional studies.	Non-systematic review studies Conference abstracts Individual studies

### Search strategy

An Information Specialist designed and conducted the search with input from the research team. Our search strategy aimed to find all systematic reviews that compared home care to alternative locations of care. The search included a mix of subject headings and keywords related to the participants (e.g., aged, senior, older, elder, geriatric) and home care (e.g., home care services, home hospitalization). The rationale for searching ‘home care’ alone was to find all reviews concerning home care and select only those that compare home care to at least one other LOC. Databases, searched from journal inception to June 2016, included: MEDLINE, the Cochrane Library, EMBASE, and Cumulative Index to Nursing and Allied Health Literature (CINAHL). The search included a mix of subject headings and keywords related to the participants (e.g., aged, senior, older, elder, geriatric) and home care (e.g., home care services, home hospitalization). Limits were applied to study designs (e.g. reviews only) and participant characteristics (e.g., not infant, child or adolescent) (Additional file 1). We also scanned the reference list of included systematic reviews for systematic review eligibility. When we were unable to obtain the full text, we emailed the first author to request the paper.

### Review selection

After removing duplicates, two reviewers independently screened citations in three phases: title, abstract, and full text. Title screening was guided by the citations’ overall relevance to this study. The eligibility criteria guided the abstract and full text screenings. Reviewers made one of three screening choices: include, exclude, or unsure. Citations were removed from further screening when both reviewers decided the article should be excluded. When at least one reviewer assigned an article to the ‘include’ or ‘unsure’ category, the article moved to the next screening stage. Inclusion and exclusion of full text articles required reviewer consensus.

### Data collection

One reviewer extracted data using a standardized pre-piloted form. A second reviewer independently verified all extracted data points for accuracy and completeness. All inconsistencies were resolved through consensus. Extracted variables included: citation information, review objectives, review methodology, review critical appraisal methods, number of studies included, number and characteristics of participants, LOCs, comparator LOCs, interventions, elder health and wellbeing outcomes, synthesis/summary of results, conclusions, and strengths/ limitations. We only extracted information from the systematic reviews that were relevant to this overview [16]. In other words, for some systematic reviews, we only examined a subset of studies (e.g., those related to the elderly or that compared LOCs).

### Data synthesis

We synthesised the data narratively using groupings of LOC comparisons that emerged from the included reviews. Pooling the data was not appropriate due to the heterogeneity in study design, population characteristics, LOCs, interventions, measures, and outcomes, within and across included systematic reviews.

### Critical appraisal of included reviews

Two raters assessed the methodological quality of included systematic reviews using the 11-item Assessing the Methodological Quality of Systematic Reviews (AMSTAR) instrument [17]. Disagreements were resolved through consensus and third person arbitration. A sensitivity analysis was conducted at the systematic review level (i.e., not primary studies). We re-examined higher quality systematic reviews that scored at or above the mean AMSTAR score.

## Results

### Review selection

**Fig. 1:**
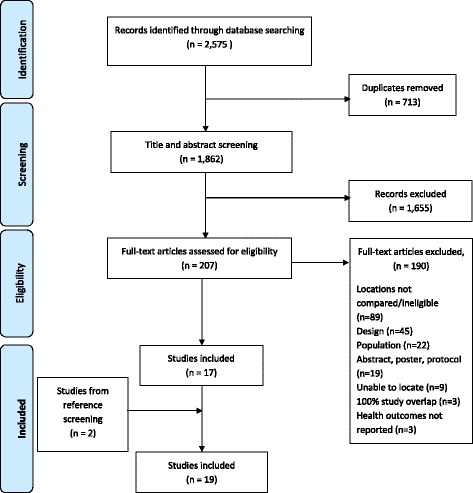
Flow chart

The search yielded 2,575 citations (Fig. 1). After removing duplicates and screening titles and abstracts, we examined 207 full texts, of which 17 were eligible for inclusion. Screening the reference lists of included systematic reviews revealed 16 potential titles, of which 2 were included. In total, 19 systematic reviews met our inclusion criteria.

### Characteristics of reviews

Included systematic reviews were published in English between 2000 and 2016 and originated from seven different countries: United Kingdom (*n* = 6), Canada (*n* = 5), Australia (*n* = 2), the Netherlands (*n* = 2), United States (*n* = 2), Switzerland (*n* = 1), and Sweden (*n* = 1) (Table 2). Three hundred and forty studies (range 1 to 110) with a reported 271,660 participants (range 96 to 108,838) were synthesized across the systematic reviews. LOC comparisons were: home with support versus independent living at home (*n* = 11 reviews), home care versus institutional care (*n* = 3 reviews), and rehabilitation at home versus other conventional rehabilitation services (*n* = 7). Data from the Mehta [18] and Fens [19] systematic reviews were included in two categories.

**Table 2:** Characteristics and scope of the included systematic reviews

Author, year, country	Aim^a^	Search strategy	No. of studies and design^a^	No. of participants^a^	AMSTAR score out of 11
Home with support versus independent living at home (*n* = 11)					
van Haastregt, 2000, [23] the Netherlands	Evaluate preventive home visits to elderly people living in the community.	Medline, Embase, and the Cochrane Controlled Trials Register	*N* = 15 All RCT	*N* = 8789	6
Markle-Reid, 2006, [21] Canada	Evaluate home-based-nursing health promotion and preventive care.	Medline, CINAHL, Cochrane Controlled Trials Register, Ageline, Health Star, PsychInfo, Sociological Abstracts, Cochrane Database of Systematic Reviews, and the Social Science Citation Index for reports of primary research	*N* = 11 (of the 12 included in review) All RCTs	*N* = 16124	6
Huss, 2008, [22] Switzerland	Evaluate preventative home visit programs aimed at maintaining health and autonomy of older adults.	MEDLINE, EMBASE, Cochrane CENTRAL database, and the Cochrane Central Register of Controlled Trials	*N* = 21 All RCTs	*N* = 14602	7
Beswick, 2010, [20] UK	Evaluate complex interventions aimed at promoting independence in older people.	Cochrane CENTRAL, MEDLINE, EMBASE, CINAHL, PsycINFO, and ISI Science and Social Science Citation Index	*N* = 110 All RCTs	*N* = 108838	4
Mehta, 2011, [18] Canada	Evaluate home physiotherapy to no physiotherapy or outpatient physiotherapy following hip fracture surgery.	MED LINE, CINAHL, EMBASE, and the Cochrane Central Register of Controlled Trials	*N* = 5 All RCTs	*N* = 329	6
Turner, 2011, [28] UK	Evaluate home modifications on the reduction of injuries.	ASSIA, British Nursing Index, CINAHL, Cochrane Library, EMBASE, ICONDA, MEDLINE, MEDLINE In-Process, Open SIGLE, Planex, RIBA-British Architectural Library Catalogue, SafetyLit, Urbadisc, Web of Science, Science Citation Index and Social Sciences Citation, Index, Conference Proceedings Citation Index	*N* = 20 (in the elder population) Cochrane review All RCTs	*N* = 9389	10
Fens, 2013, [19], the Netherlands	Evaluate multidisciplinary care delivered to stroke patients living in the community after discharge from hospital or inpatient rehabilitation.	PubMed, EMBASE, CINAHl and the Cochrane Library	*N* = 4 (of 14 included) All RCTs	*N* = 774	5
You, 2013 [25] Australia	Evaluate case management in community aged care (CMCAC) interventions on service use and costs.	Web of Science, Scopus, Medline, CINAHL (EBSCO) and PsycINFO (CSA). Google Scholar identified studies unavailable in these databases	*N* = 21 RCT, cluster RCT (*n* = 16), comparative observational (*n* = 5)	*N* = 44012	5
Bryant-Lukosius, 2015, [24] Canada	Evaluate clinical and cost effectiveness of clinical nurse specialists providing transitional care.	CINAHL, EMBASE, Global Health, HealthStar, Medline, Allied and Complementary Medicine Database (AMED), Cochrane Library Database of Systematic Reviews and Controlled Trials Register, Database of Abstracts of Reviews of Effects, Health Economics Evaluation Database, and Web of Science	*N* = 5 (of 13 included) All RCTs	*N* = 964	8
Reilly, 2015 [26] UK	Evaluate case management approaches to home support for people with dementia.	ALOIS, the Specialised Register of the Cochrane Dementia and Cognitive Improvement Group, The Cochrane Library, MEDLINE, EMBASE, PsycINFO, CINAHL, LILACS, Web of Science, Campbell Collaboration/SORO database and the Specialised Register of the Cochrane Effective Practice and Organisation of Care Group	*N* = 13 All RCTs	*N* = 9615	10
Liu, 2016, [27] Canada	Evaluate effectiveness of smart homes and home health-monitoring technologies for older adults with complex needs.	Scopus, PubMed, Cinahl, IEEE Explore, ISI Web of Sciences, and ACM Digital Library	*N* = 48 RCTs and other designs (specifics NR)	*N* = 11375	8
Home care versus institutional care (*n* = 3)					
Mottram, 2002, [7] UK	Compare outcomes of long term home care versus institutional care for functionally dependent older people.	Cochrane Effective Practice and Organization of Care Group (EPOC), the Cochrane Controlled Trials Register, MEDLINE, EMBASE, Best Evidence, Ageline, Cinahl, EconLit, PsycInfo, NTIS, Scisearch, Sigle.	*N* = 1 Cochrane Review RCT	*N* = 112	9
Gomes, 2013, [30] UK	Evaluate home palliative care services, odds of dying at home, and the clinical effectiveness of home palliative care services.	Cochrane Central Register of Controlled Trials, EMBASE, MEDLINE, Cochrane Pain, Palliative and Supportive Care Trials Register, Cochrane Effective Practice and Organization of Care Trials Register, CINAHL, EURONHEED, PsycINFO, Cochrane Database of Systematic Reviews, Database of Abstracts of Reviews of Effectiveness, Health Technology Assessment Database, NHS Economic Evaluation Database	*N* = 23 RCT (*n* = 16) Controlled trial, controlled before and after, interrupted time series with nested controlled before and after (*n* = 7)	*N* = 41603 *n* = 37561 (elderly) *n* = 4042 (caregivers)	10
Wysocki, 2015, [29] USA	Evaluate outcomes of older adults receiving home and community-based support versus those living in nursing homes.	Medline (via Ovid), AgeLine	*N* = 14 Longitudinal RCTs and quasi-experimental observational designs (specifics NR)	NR	6
Rehabilitation at home versus conventional rehabilitation care (*n* = 7)					
Britton, 2000, [32] Sweden	Evaluate post-stroke home rehabilitation compared to conventional alternatives.	MEDLINE, Cochrane Library, Cinahl, Econlit, ArbSpriline, ABI Inform, and Sociological Abstracts.	*N* = 7 RCT (*n* = 6), controlled trial (*n* = 1)	*N* = 1487	6
Toussant, 2005, [34] United States	Evaluate physical therapy in the management of hip fractures that were surgically treated.	PubMed, ProQuest, EBSCO.	*N* = 3 (of 15 included) RCT, blocked RCT equivalence trial	*N* = 451	4
Hillier, 2010, [33] Australia	Evaluate stroke rehabilitation delivered in the home compared to an outpatient clinic or day hospital setting.	Cochrane library, Ovid (Medline, AMED, Embase, Ageline), Ebsco (Cinahl) and PEDro.	*N* = 6 (of 15 publications) All RCTs	*N* = 1170	6
Mehta, 2011, [18]	As above	As above	As above	As above	As above
Allen, 2012, [35] Canada	Evaluate rehabilitation practices for patients with dementia who have had a hip fracture	Medline, CINAHL, Cochrane, Embase, PEDro, PsychINFO, Web of Science, and Scopus.	*N* = 1 (of 13 included) Prospective longitudinal cohort	*N* = 96	4
Fens, [19]	As above	As above	*N* = 5 (of 14 included in review) All RCTs	*N* = 1029	As above
Brown, 2015, [31] UK	Evaluate multidisciplinary rehabilitation services in medical day hospitals on older patients’ health outcomes.	Cochrane Effective Practice and Organisation of Care Group Register of Studies, CENTRAL, MEDLINE via Ovid, EMBASE via Ovid and CINAHL via EbscoHost	*N* = 7 (of 16) All RCTs	*N* = 901	10

*AMSTAR* = Assessing the Methodological Quality of Systematic Reviews, *RCT* Randomized control trial, *NR* not reported

^a^ Aims reported here are those relevant to this overview. When appropriate, only the subset of studies specific to the eligibility of this overview are reported

### Summary of findings

#### Home with support versus independent living at home (*n* = 11 reviews)

Compared to usual care, typically defined as independent living at home, seven reviews favoured home with support, three found no difference, and one reported insufficient evidence (Tables 3 and 4). Home support by an interdisciplinary team reduced nursing home and hospital admissions, decreased falls, and improved physical function [20]. Two reviews found that preventative home visits for community dwelling elders improved health and functional status, mortality rates, and delayed hospitalization and nursing home use [21, 22]. However, another review found no difference in health outcomes due to preventative home visits [23]. Transitional support provided by clinical nurse specialists reduced re-hospitalizations [24]. Two reviews examined case management interventions (i.e., collaborative patient care to meet patients’ holistic needs). One found delayed onset of nursing home placement and decreased nursing home admissions, length of stay, and community care service use [25]. The other found that case management approaches for people with dementia improved cognitive status and decreased institutionalization and caregiver outcomes at certain time points [26]. One review concluded that home health monitoring technologies reduced undesired health outcomes for the elderly [27]. Another review found limited evidence for the effectiveness of multidisciplinary care for stroke patients at home compared to usual care [19]. Mehta [18] found no difference between receiving home physiotherapy for hip fractures compared to no physiotherapy. Finally, a Cochrane review found little high-grade evidence that physical modifications to the home environment affected the likelihood of sustaining an injury in the home [28].

**Table 3:** Findings of included systematic reviews

Author, year	PICO (Population, intervention, comparison, outcome)	Summary of findings	Appraisal approach & quality of the evidence^a^	Favored location of care
Home with support versus independent living at home (*n* = 11)				
van Haastregt, 2000 [23]	P: Elders 65+ years living in the community I: Preventive home visitation programs provided by a nurse or equivalent C: Usual care O: Health indicators	Little evidence exists in favor of the effectiveness of preventive home visits to elderly people living in the community. None of the trials reported negative effects.	Cochrane Collaboration (1997) Study scores ranges from 20% to 71% with a mean of 54%.	No difference
Markle-Reid, 2006 [21]	P: Elders 65+ years living at home and eligible for home care services I: Preventive home visitation programs provided by a nurse or equivalent C: Usual care O: Health indicators	Home visit interventions by nurses improved health and functional status, mortality rates, and use of hospitalization and nursing homes.	Tool developed by Jadad et al., 1996. Trials fulfilled most effectiveness criteria. Shortcomings were randomization, blinding and follow-up.	Home with support
Huss, 2008 [22]	P: Elders 70+ years living in the community I: Multidimensional preventive home visitation programs C: Usual care O: Health indicators	Multidimensional preventive home visits may reduce disability burden among older adults when based on multidimensional assessment with clinical examination.	Evaluated concealment of allocation, and blinding of staff. 50% reported adequate blinding; 29% had adequate concealment.	Home with support
Beswick, 2010, [20]	P: Elders 65 + years living at home or preparing for hospital discharge to home I: Community-based multifactorial interventions with preventive strategies and subsequent active management C: Usual care O: Indicators of independence	Overall benefit of complex community interventions in helping older people to live at home and maintain independence.	Evaluated loss to follow-up and randomization. Specifics not reported. Same effect sizes in studies of different quality.	Home with support
Mehta, 2011, [18]	P: Patients who have had surgery for a hip fracture I: Home physiotherapy C: No physiotherapy and outpatient physiotherapy O: Indicators of health, quality of life, performance-based indicators	Home physiotherapy was better than no physiotherapy care for improving patient-reported health-related quality of life.	Cochrane risk of bias tool Overall moderate to low quality evidence	No difference
Turner, 2011 [28]	P: Older people living at home I: Modification of physical hazards in the home and related components (e.g., education) C: Usual care O: Injury rate	The effect of home modification on falls was either inseparable or insignificant.	EPOC checklist Inclusion criteria limited to higher quality RCTs.	Evidence was inseparable or insignificant
Fens, 2013, [19]	P: Post-stroke older patients discharged home I: Multidisciplinary care including home assessment, assessment with follow-up care, education. C: Usual Care O: Indicators of health and well-being	Limited evidence for the effectiveness of multidisciplinary care for stroke patients being discharged home compared to usual care.	CONSORT Studies ranged from 35% to 62%, with a mean of 50%	No difference
You, 2013 [25]	P: Community- dwelling frail elders aged 65+ years I: Independent case management interventions applied in the community C: Usual care O: Health service use	Moderate evidence that case management interventions can improve clients’ use of some community care services and delays nursing home placement, reduces nursing home admission, and shortens length of nursing home stay.	Used a checklist informed by evidence. Moderate and lower quality studies.	Home with support
Bryant-Lukosius, 2015 [24]	P: Patients receiving care (subgroup analysis of elderly) I: Transitional support from a clinical nurse specialist C: Usual care O: Health system utilization, patient health outcomes, caregiver outcomes	Evidence that clinical nurse specialist transitional support reduced re-hospitalizations and improved caregiver depression. The effects on other outcomes were less clear.	Modified Cochrane risk of bias tool GRADE Moderate risk (n-3); high risk (*n* = 2) Low-quality evidence where GRADE could be applied	Home with support
Reilly, 2015 [26]	P: People with dementia living in the community and their carers I: Case management (planning and coordination of care) C: Usual care O: Health and health resource indicators	Some evidence that case management is beneficial for improving some outcomes at certain time points, both in the person with dementia and in their caregiver.	Cochrane risk of bias tool Low to moderate overall risk of bias	Home with support
Liu, 2016 [27]	P: Adults 60+ years with complex needs I: Supportive care environment at home, including technology use C: Usual care O: Aging in place indicators, technology readiness	Home health monitoring technologies reduce some negative health outcomes for the elders, however, elder technological readiness is low.	PEDro scale and Sackett criteria Quality of the individual studies were unclear	Home with support
Home care versus institutional care (*n* = 3)				
Mottram, 2002 [7]	P: Older adults in need of care services I: Enhanced long-term home-care services C: Institutional long-term care O: Health indicators	Insufficient evidence to determine whether dependent older people fare better at home with care services compared to living in institutional long-term care.	Descriptive Study rated as small and of poor methodological quality	Insufficient evidence
Gomes, 2013 [30]	P: Older adults with a life-limiting chronic disease I: Home palliative care; reinforced home palliative care C: Usual care (varied depending on local context) O: Death at home	Home palliative care increases the chance of dying at home and reduces symptom burden, particularly for patients with cancer.	EPOC checklist Inclusion criteria limited to higher quality RCTs. 6/16 RCTs were high quality; 0/4 controlled clinical trials were high quality	Home with support
Wysocki, 2015, [29]	P: Adults aged 60+ years I: Home and community based care C: Institutional care O: Function, cognition, mental health, acute care use, mortality.	No difference between most outcomes for LOC. Insufficient evidence to draw conclusions about preferred LOC.	Agency for Healthcare Research and Quality Low methodological quality	Insufficient evidence
Rehabilitation at home versus conventional rehabilitation (*n* = 7)				
Britton, 2000, [32]	P: Elderly participants requiring rehabilitation services after stroke I: Home rehabilitation started after acute hospital stay. C: Conventional rehabilitation O: Health and functional indicators	No statistically significant differences in outcomes between home rehabilitation and conventional care for activities of daily living, depression, quality of life, social activities, stress, satisfaction, depression, and quality of life for family members.	Quality factors (Drummond & Jefferson, 1996) Moderate to high	No difference
Toussant, 2005, [34]	P: Elderly with a sustained a hip fracture I: Home based physical therapy rehabilitation to manage surgically treated hip fractures C: Conventional rehabilitation O: Health and functional indicators	Home-based rehabilitation programs involving physical therapy are as beneficial as intensive hospital rehabilitation programs.	Sackett’s rules of evidence and grades (1989) Grade B evidence (supported by at least 1 small RCT with low risk of false positive/ negative)	No difference
Hillier, 2010 [33]	P: Community-dwelling participants within 1-year post-stroke I: Stroke rehabilitation delivered at home C: Stroke rehabilitation delivered in a center O: Independence in function	Home-based rehabilitation is superior to centre-based for functional benefits in the early period post-discharge from an inpatient setting. There is conflicting evidence that the results remain in favour of home-based long-term (6 months)	PEDro Criteria Range 7 to 9 of 11; mean = 9/11	Home rehabilitation
Mehta, 2011, [18]	As above	Home physiotherapy was similar to outpatient physiotherapy in improving patient-reported health-related quality of life. Performance-based outcomes were marginally better following outpatient physiotherapy compared with home physiotherapy 3 and 6 months after surgery. Due to the poor methodological quality of included studies, the authors concluded insufficient evidence to recommend one setting over another.	As above	Insufficient evidence
Allen, 2012, [35]	P: Patients with hip-fracture and dementia I: Home rehabilitation C: Conventional rehabilitation O: Indicators of function	Comparable functional recovery outcomes in patients with dementia recovering from hip fracture across locations compared.	Newcastle–Ottawa Quality Assessment Scale for Cohort Studies Studies scored 5, 7 of 8	No difference
Fens, 2013, [19]	P: Post-stroke older patients discharged home I: Home rehabilitation C: Conventional rehabilitation O: Indicators of function	No differences between rehabilitation at home versus conventional settings.	CONSORT Studies ranged from 54% to 73%, with a mean of 60%	No difference
Brown, 2015 [31]	P: Older people I: Medical day hospitals C: Domiciliary care O: Indicators of health	No difference in health outcomes between elders that receive home rehabilitation services compared to those who receive rehabilitation services at a medical day hospital.	Cochrane risk of bias tool Low to moderate overall risk of bias Overall low quality evidence	No difference

^a^ Reported by the authors of the systematics review

**Table 4:** Health outcomes reported by included systematic reviews

Author, year	Function/independence	Satisfaction	Mortality	Healthcare usage	Institutional care	Caregiver outcomes	Falls	Cognition	Symptom burden	Quality of life	Mental health	Death at home
Home with support versus independent living at home (*n* = 11)												
van Haastregt, 2000 [23]	ND		ND		ND		ND				ND	
Markle-Reid, 2006 [21]	+		+	+	+	+						
Huss, 2008 [22]	+		+ (for lowest age quartile)		ND							
Beswick, 2010 [20]	Mixed		ND		+		+					
Mehta, 2011, [18]	ND									+ (at 3 months) + (at 6 months)		
Turner, 2011 [28]							ND					
Fens, 2013, [19]	ND									Mixed		
You, 2013 [25]				+	+							
Bryant-Lukosius, 2015 [24]		ND	ND	+		+						
Reilly, 2015 [26]			ND	ND	+	+ (at 6 months)		+		ND		
Liu, 2015 [27]	+						ND	+		ND		
Home care versus institutional care (*n* = 3)												
Mottram, 2002 [7]	ND			ND						ND	ND	
Gomes, 2013 [30]									+			+
Wysocki, 2015, [29]	ND	ND		ND				ND			ND	
Rehabilitation at home versus conventional rehabilitation services (*n* = 7)												
Britton, 2000, [32]	ND									ND		
Toussant, 2005, [34]	ND										ND	
Hillier, 2010, [33]	+						+					
Mehta, 2011, [18]	ND									+ (at 3 months) + (at 6 months)		
Allen, 2012, [35]	ND							ND		ND		
Fens, 2013, [19]	ND									ND		
Brown, 2015 [31]	ND		ND	IE	ND	ND						

Referencing home with support, community dwelling, or rehabilitation at home, respectively

(+) = favourable, (-) = unfavourable, *ND* no difference, *IE* insufficient evidence

#### Home care versus institutional care (*n* = 3 reviews)

Three reviews compared home care to institutional care. Although each review differed in their purpose and scope, two found insufficient evidence, and one review favoured home care (Tables 3 and 4). A Cochrane review that examined the health outcomes of elders receiving home care compared to institutional care found only one randomized controlled trial and concluded that there was insufficient evidence to make recommendations about alternatives to institutional care for the elderly [7]. Similarly, a review that examined various home care options compared to nursing homes for care-dependent elders found no significant differences for most health outcomes [29]. Another Cochrane review found that elders who received home palliative care compared to institutional palliative care were more likely to die at home and experience less symptom burden, particularly for patients with cancer [30].

#### Home rehabilitation versus conventional rehabilitation care (*n* = 7 reviews)

Within this category, five reviews reported no differences between home and conventional rehabilitation care, one concluded insufficient evidence to make a recommendation, and one reported that home rehabilitation was superior to conventional options in the short-term (Tables 3 and 4). Specifically, one Cochrane review included seven studies that compared home-based rehabilitation to medical day hospital rehabilitation for community dwelling elders [31]. Findings showed no difference between these settings on health outcomes. Three reviews examined post-stroke rehabilitation. In one review, there were no significant differences between home and conventional rehabilitation for activities of daily living, depression, quality of life, and social activities [32]. Pooled evidence from the other review suggested that home-based rehabilitation, provided between 6 and 12 weeks after discharge from inpatient care, might yield superior functional benefit and satisfaction; however, benefits were less clear after 6 months [33]. The third review showed no difference between home and conventional rehabilitation for outcomes related to activities of daily living and quality of life [19].

Three reviews examined rehabilitation after hip fractures. One review found no difference between home and outpatient physiotherapy for patient-reported health-related quality of life, but suggested that performance-based outcomes might be marginally better following outpatient services [18]. However, due to the low quality of the primary studies the authors concluded that there was insufficient evidence to recommend one service delivery model over the other. A similar review found that home-based rehabilitation programs involving physical therapy for hip fractures were as beneficial as hospital rehabilitation for patients who had not lost many functional abilities prior to the fracture [34]. The other review examined elders with dementia and hip fractures and found no differences in health outcomes between the settings [35].

### Critical appraisal with sensitivity analysis

Based on the AMSTAR ratings, the average quality of included systematic reviews was moderate, with a mean AMSTAR score of 7 of 11 (median = 6; range = 4 to 10) (Table 5). No systematic reviews met the criteria of disclosure of financial interest (Item 11) because none reported funding/support of each included study. We re-examined systematic reviews scoring at or above the mean. In the home with support versus independent living at home category, 5 of 11 reviews were of higher quality. Four reviews, including one Cochrane, which examined preventative home visits, transitional support, case management, and home health technologies, concluded that home with support was superior to independent living at home [22, 24, 26, 27]. The other, a Cochrane review, concluded that inseparable or insignificant results for home modification to reduce falls [28].

**Table 5:**
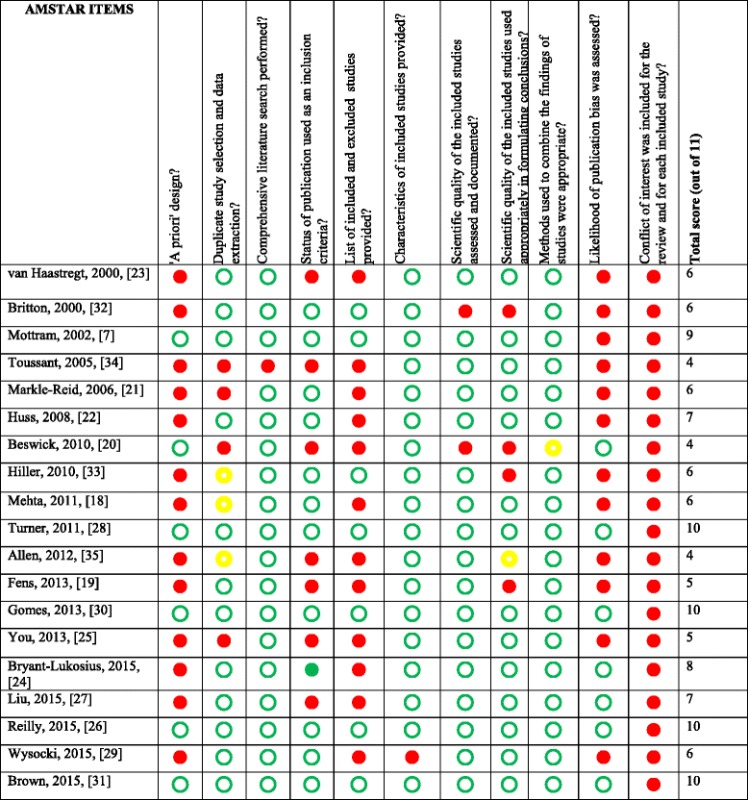
Authors’ judgements about each risk of bias item of included systematic reviews

= yes

= no

= unsure

Two of three reviews in the home care versus institutional care category were high quality (AMSTAR ratings were 9 and 10 of 11, both Cochrane reviews). The findings, however, were mixed: one review concluded that there was insufficient evidence to determine whether elders fare better with home care compared to institutional care [7] and the other supported home palliative care over institutional palliative care to improve the likelihood of dying at home and decreasing symptom burden [30]. Only the Brown et al., [31] Cochrane review was of high enough quality (AMSTAR score of 10/11) to be included in the home versus conventional rehabilitation category. This review found no significant differences in elder health outcomes between those who received home rehabilitation compared to those who attended medical day hospitals for rehabilitation services.

## Discussion

We synthesized systematic reviews evaluating the impact of home care versus alternative LOCs on elder health outcomes. Overall, we found 19 eligible systematic reviews covering 340 studies and 271,660 participants. Reviews originated from seven industrialised countries that are known to be experiencing rapid growth among their elder population. Categories of comparisons included: home with supports versus independent living at home, home care versus institutional care, and home rehabilitation versus conventional rehabilitation. Most reviews favoured home with support to independent living at home. Results for home care versus institutional care were mixed. Most reviews found no differences in health outcomes between rehabilitation at home versus conventional rehabilitation services. The quality of included systematic reviews was moderate. Our results lead us to make the following observations.

First, as aging in a desirable LOC may contribute to overall health and wellbeing in the late-life period [36, 37], we were not surprised to find several reviews that examined LOC as a function of elder health and wellbeing. Many elderly people prefer and choose to age at home [38–42]. Healthcare organizations and policy makers are increasingly challenged to better support a shift from institutionalized long-term care to support elders to remain in their community [43]. The included reviews indicate a growing literature of interventions designed to promote health, function and independent living among community dwelling elders. Yet, many older adults anticipate moving, often to a more institutionalized location. Even those who do not anticipate moving might be compelled to move due to unforeseen life changes (e.g. loss of a spouse, illness) [40, 44]. As life expectancy increases and more people in late life suffer from multimorbidity, their relocation will represent a significant market for new residential options beyond the limited choices of private nursing homes or public long-term care facilities [45]. Therefore, an improved understanding of the role of long-term LOCs on elders’ health and wellbeing is central to any strategy aimed at fostering elders’ quality of life [46].

Second, the impact of home care compared to institutional care on elder health outcomes was less clear. The Mottram et al. [7] and Wysocki et al. [29] reviews had similar PICOS questions, and despite the 13-year publication gap and new research, both concluded that there was insufficient evidence to draw firm conclusions. Similarly, most systematic reviews that examined location of rehabilitation services and elder health outcomes found no differences or insufficient evidence. Inconclusive findings suggest that informed patient preferences and individual needs should guide elders’ decision to move from home to an alternative LOC. Examination of LOC options and preferences should also incorporate contextual factors, such as the quality of available services or other interventions. Consistent the ecological model of aging and person-environment fit, elders’ specific needs (e.g., mobility, cognitive, social) must be evaluated and matched to available LOC options with appropriate interventions or services to address those needs.

Third, our results highlight that most home care research focuses on few LOC comparisons. Further, most systematic reviews did not compare across multiple potential LOCs. When examined individually, each comparison provided insight regarding potential outcomes of one LOC option compared to the other. However, it was not possible to integrate the findings to determine the best LOC based on elder health outcomes. Although we did not examine the primary studies, our overview identifies a need for better research targeting long-term LOC options for the late-life years. Improved research and additional investment will translate into more rigorous methods and generate a more complete understanding of the impact of LOCs on elders’ health.

Our findings should be interpreted within the context of its limitations. First, our search strategy focused on home care, thus terms such as ‘day hospital’ and ‘assisted living’ were not included. Also, we did not search the grey literature. Therefore, we might have missed eligible systematic reviews. Second, several factors made this literature challenging to synthesize. We found considerable heterogeneity within and across included systematic reviews regarding population characteristics, locations considered, measures used, and outcomes reported, precluding firm conclusions. LOCs were also poorly and inconsistently described in the literature. Development of an accepted taxonomy for LOCs would help advancement in this field. Third, the study designs of the included reviews made direct comparisons for LOCs difficult and we noted a risk of indication bias (elders’ health status correlates with the intervention and is a risk indicator for the outcome) within several of the primary studies. Causal relationships between LOCs and elder health outcomes could not be measured. Instead, this overview provides a broad summary of the state of knowledge regarding elder home care compared to alternative LOCs and elder health outcomes. Fourth, there was some primary study overlap across the systematic reviews, potentially over-representing certain findings. However, the purpose of an overview of systematic reviews is to provide a high level description of the state of the evidence [16]. To offset this limitation, we excluded studies with 100% of primary study overlap. Finally, new and relevant studies might have been excluded if they were not yet included in a systematic review.

## Conclusions

Results from this overview of systematic reviews, which evaluated the impact of home care compared to alternative long-term LOCs on elder health outcomes, suggests that home interventions and/or supports that promote elder health and independence might be effective in helping elders age at home. However, we are unable to make recommendations regarding the impact of other LOCs compared to home on elders’ health and wellbeing. Without a more robust evidence base, elders considering moving from home will be unable to make evidence informed decisions about which long-term alternative LOC to choose that fits best with their needs and preferences.

## Additional file

Additional file 1: Search strategy for Medline. (DOCX 13 kb)

## Abbreviation

LOC: Location of care
