# Supplementary material for: Impact of home care versus alternative locations of care on elder health outcomes: an overview of systematic reviews
Source: BMC Geriatr. 2017 Jan 14;17:20. doi: 10.1186/s12877-016-0395-y (PMC5237488; doi:10.1186/s12877-016-0395-y)
Supplement: Additional file 1: — Search strategy for Medline. (DOCX 13 kb) [file 12877_2016_395_MOESM1_ESM.docx]

**Additional file 1 – Search strategy for Medline (OVID)**

**MedLine (OVID)**

|  | 1 | exp geriatric assessment/ |
| --- | --- | --- |
|  | 2 | exp aged/ |
|  | 3 | (aged 65 and over).mp. |
|  | 4 | (aged or senior$ or elder$ or older).mp. |
|  | 5 | geriatric*.mp. |
|  | 6 | (old* adj person*).mp. |
|  | 7 | (senior* adj citizen*).mp. |
|  | 8 | (old adj people*).mp. |
|  | 9 | exp Aging/ or exp Retirement/ or retire*.mp. |
|  | 10 | or/1-9 |
|  | 11 | Home-based versus hospital-based.tw. |
|  | 12 | Home hospitalization.tw. |
|  | 13 | exp Home Care Services/ |
|  | 14 | Home Care Services, Hospital-Based/ |
|  | 15 | Community Health Nursing/ |
|  | 16 | exp home nursing/ |
|  | 17 | ((home or homebased or domicil$ or homecare) adj3 treat$).tw. |
|  | 18 | ((home or homebased or domicil$) adj3 (care$ or caring)).tw. |
|  | 19 | ((home or homebased or domicil$ or homecare) adj3 healthcar$).tw. |
|  | 20 | or/11-19 |
|  | 21 | (child* or infant* or adolescent*).mp. |
|  | 22 | (((comprehensive* or systematic*) adj3 (bibliographic* or review* or literature)) or (meta-analy* or metaanaly* or "research synthesis" or ((information or data) adj3 synthesis) or (data adj2 extract*))).ti,ab. or (cinahl or (cochrane adj3 trial*) or embase or medline or psyclit or (psycinfo not "psycinfo database") or pubmed or scopus or "sociological abstracts" or "web of science").ab. or "cochrane database of systematic reviews".jn. or ((review adj5 (rationale or evidence)).ti,ab. and review.pt.) or meta-analysis as topic/ or Meta-Analysis.pt. |
|  | 23 | (child* or infant* or adolescent*).mp. |
|  | 24 | 10 and 20 |
|  | 25 | 24 not 21 |
|  | 26 | 25 and 22 [Reviews] |
|  | 27 | limit 26 to ed=20141119-20160701 |
